# Demographic Differences Among US Department of Veterans Affairs Patients Referred for Genetic Consultation to a Centralized VA Telehealth Program, VA Medical Centers, or the Community

**DOI:** 10.1001/jamanetworkopen.2022.6687

**Published:** 2022-04-11

**Authors:** Maren T. Scheuner, Alexis K. Huynh, Catherine Chanfreau-Coffinier, Barbara Lerner, Alicia R. Gable, Martin Lee, Alissa Simon, Randall Coeshott, Alison B. Hamilton, Olga V. Patterson, Scott DuVall, Marcia M. Russell

**Affiliations:** 1 San Francisco Veterans Affairs Health Care System, San Francisco, California; 2 University of California, San Francisco, School of Medicine, San Francisco; 3 Center for the Study of Healthcare Innovation, Implementation, and Policy, Veterans Affairs Greater Los Angeles Health Care System, Los Angeles, California; 4 Veterans Affairs Informatics and Computing Infrastructure, Salt Lake City, Utah; 5 Veterans Affairs Boston Health Care System, Boston, Massachusetts; 6 University of California Los Angeles Fielding School of Public Health, Los Angeles; 7 David Geffen School of Medicine at University of California Los Angeles, Los Angeles; 8 Division of Epidemiology, University of Utah, Salt Lake City

## Abstract

**Importance:**

Telehealth enables access to genetics clinicians, but impact on care coordination is unknown.

**Objective:**

To assess care coordination and equity of genetic care delivered by centralized telehealth and traditional genetic care models.

**Design, Setting, and Participants:**

This cross-sectional study included patients referred for genetic consultation from 2010 to 2017 with 2 years of follow-up in the US Department of Veterans Affairs (VA) health care system. Patients were excluded if they were referred for research, cytogenetic, or infectious disease testing, or if their care model could not be determined.

**Exposures:**

Genetic care models, which included VA-telehealth (ie, a centralized team of genetic counselors serving VA facilities nationwide), VA-traditional (ie, a regional service by clinical geneticists and genetic counselors), and non-VA care (ie, community care purchased by the VA).

**Main Outcomes and Measures:**

Multivariate regression models were used to assess associations between patient and consultation characteristics and the type of genetic care model referral; consultation completion; and having 0, 1, or 2 or more cancer surveillance (eg, colonoscopy) and risk-reducing procedures (eg, bilateral mastectomy) within 2 years following referral.

**Results:**

In this study, 24 778 patients with genetics referrals were identified, including 12 671 women (51.1%), 13 193 patients aged 50 years or older (53.2%), 15 639 White patients (63.1%), and 15 438 patients with cancer-related referrals (62.3%). The VA-telehealth model received 14 580 of the 24 778 consultations (58.8%). Asian patients, American Indian or Alaskan Native patients, and Hawaiian or Pacific Islander patients were less likely to be referred to VA-telehealth than White patients (OR, 0.54; 95% CI, 0.35-0.84) compared with the VA-traditional model. Completing consultations was less likely with non-VA care than the VA-traditional model (OR, 0.45; 95% CI, 0.35-0.57); there were no differences in completing consultations between the VA models. Black patients were less likely to complete consultations than White patients (OR, 0.84; 95% CI, 0.76-0.93), but only if referred to the VA-telehealth model. Patients were more likely to have multiple cancer preventive procedures if they completed their consultations (OR, 1.55; 95% CI, 1.40-1.72) but only if their consultations were completed with the VA-traditional model.

**Conclusions and Relevance:**

In this cross-sectional study, the VA-telehealth model was associated with improved access to genetics clinicians but also with exacerbated health care disparities and hindered care coordination. Addressing structural barriers and the needs and preferences of vulnerable subpopulations may complement the centralized telehealth approach, improve care coordination, and help mitigate health care disparities.

## Key Points

**Question:**

How is a centralized telehealth model associated with care coordination and equity of genetic services delivery?

**Findings:**

In this national cross-sectional study of 24 778 adult patients with genetic referrals, certain racial and ethnic groups were significantly less likely to be referred to a centralized telehealth model than traditional genetic services, and completing consultations was significantly less likely for Black patients referred to the telehealth model. Patients were more likely to have multiple cancer preventive procedures if they completed their consultations but only if completed with traditional genetic services.

**Meaning:**

These findings suggest that, while a centralized telehealth model may improve access to genetics clinicians, care coordination may be compromised, and health care disparities may be exacerbated compared with a traditional care model.

## Teaser

This cross-sectional study assesses care coordination and equity of genetic care delivered by centralized telehealth and traditional genetic care models for US Department of Veteran Affairs patients.

## Introduction

Genetic information can transform health care and improve health outcomes through better diagnosis, prognosis, risk assessment, prevention, and targeted treatments. Clinicians with training in medical genetics have expertise in the clinical application of genetic information.^1^ However, medical genetics is a specialty that is difficult to access because of insufficient numbers of clinicians to meet the demand, with most located in academic, metropolitan settings.^1,2^ New practice models are needed to realize the potential of genomic medicine and to ensure all Americans have access to genetic services.^3^

Telehealth enables the delivery of health-related services across long distances, increasing access to care and the reach of specialized clinical expertise, like genetics.^3^ The use of telehealth by clinical geneticists increased from 16% in 2015 to 33% in 2019, and telehealth became the predominant delivery mode during the COVID-19 pandemic.^3,4,5,6^ However, evidence addressing the influence of telehealth on care coordination and health care equity is lacking.^7^

The Department of Veterans Affairs (VA) oversees the largest integrated health care system in the US,^8^ and health care is provided via telehealth at more than 900 sites across the country in over 50 areas of specialty care.^9^ In the VA, a national Virtual Health Care System is being considered to improve accessibility and convenience of care for certain health care services.^10^ Genetic services became widely available within the VA in 2010 when the VA Genomic Medicine Service launched a model program that embodies the goals of the VA’s Virtual Health Care System. This centralized program is staffed by a team of genetic counselors that serves about 80 VA facilities nationwide via telehealth. Before establishing this service, very little genetic care was provided within the VA. The earliest traditional programs were based in Houston, Texas, and Los Angeles, California, with few referrals before 2010. Today, 6 traditional genetics programs exist comprising small teams of clinical geneticists and genetic counselors serving patients at 1 or multiple VA facilities within a region via multiple delivery modes.^11,12^ Non-VA care purchased in the community is also available if there are long wait times or long geographic distances to VA clinicians or the required care is not available within the VA. In 2010, standardized community care referrals for genetic consultation were established. The purpose of this study was to assess care coordination and equity in the delivery of genetic care for the care models available to VA patients (ie, VA-traditional, VA-telehealth, and non-VA care) since their inception.

## Methods

We conducted a cross-sectional study of patients referred to the 3 genetic care models in the VA. The study was informed by the Care Coordination Measurement Framework,^13^ and we used the Health Equity Implementation Framework to understand potential health care inequities.^14^ The VA central institutional review board approved all study activities, and informed consent was waived because the study was deemed minimal risk. This study followed the Strengthening the Reporting of Observational Studies in Epidemiology (STROBE) reporting guideline.

We used the VA Corporate Data Warehouse^15^ to identify and characterize patients with a genetic consultation referral from January 1, 2010, to December 31, 2017. SQL scripts were used to extract data from each consultation regarding patient characteristics (ie, gender, age, self-reported race and ethnicity, marital status, service-connected disability, and health insurance) and consultation characteristics (ie, year of consultation, consultation status, referral reason, referring and receiving sites, clinician type, and mode of delivery). Natural Language Processing was used to characterize mostly text-based data describing the referral reasons into 15 categories: allergy-immunology; congenital disorder (including chromosomal disorders); cancer; cardiovascular (CVD) or connective tissue disorder (CTD); endocrine or metabolic; gastrointestinal (GI) or polyposis; hematology; nephrology or urology; neurology or psychiatry; personal use (eg, ancestry, paternity); pharmacogenetics or exposures; pulmonary; reproductive concerns; rheumatology or autoimmune disorders; and not specified (eg, genetic counseling, genetic screening, positive genetic finding) (eAppendix in the Supplement). We excluded patients if their genetic consultation was submitted for research, cytogenetic, or infectious disease testing, or if the type of genetic service model could not be determined. Clinician type (eg, clinical geneticist, genetic counselor) was extracted from signature blocks in the consultation notes when available. Mode of delivery (in-person, video-telehealth, telephone, e-consultation) for the visit was extracted from administrative codes and note titles; when unavailable, the note was searched for keywords describing the delivery mode. Cancer surveillance (eg, mammogram, colonoscopy) and risk-reducing procedures (eg, colectomy, bilateral mastectomy) occurring within the VA setting within 2 years following the genetics referral were identified using current procedural terminology codes.

### Statistical Analysis

We used multivariate regression models to assess associations between patient and consultation characteristics and the following outcomes: (1) genetic care model referral (multinomial logistic regression); (2) genetic consultation completion (binary logistic regression); and (3) having 0, 1, or 2 or more cancer surveillance and risk-reducing procedures within 2 years following the genetics referral (ordinal logistic regression). Using interaction terms, we examined the moderating effects of age and race or ethnicity on gender (outcomes 1-3), genetic care model on age, gender, and race or ethnicity (outcomes 2 and 3), and genetic care model on consultation completion status (outcome 3). Only 1.4% of the participants were excluded from the analyses because of missing data (eTable 1 in the Supplement). We obtained robust variance estimates for all multivariate models to account for the clustering of patients within referring facilities (VA medical centers and their associated community-based outpatient clinics).^16,17^ Statistical significance was set at *P* < .05.

## Results

### Patient Characteristics

We identified 24 778 patients with a genetic consultation, including 12 671 women (51.1%), 13 193 patients aged 50 years or older (53.2%), and 15 639 White individuals 63.1% (Table 1). Patients were referred from 114 VA facilities from January 1, 2010, to December 31, 2017, and 14 580 of 24 778 patients (58.8%) were referred to the VA-telehealth model. There were inconsequential differences in proportions of the demographic characteristics of patients referred from the source populations of the VA-telehealth (n = 7 058 074) and VA-traditional (n = 1 050 855) models (eTable 2 in the Supplement).

**Table 1.** Patient Characteristics by Type of Genetic Health Care Model

Patient characteristics	Patient, No. (%)			
	VA-traditional model (n = 6775)	VA-telehealth model (n = 14 580)	Non-VA care (n = 3423	Total (n = 24 778)
Age, mean (SD), y	50.7 (15.3)	50.8 (14.8)	49.4 (14.2)	50.6 (14.9)
Age groups^a^				
<50	3102 (45.8)	6756 (46.3)	1727 (50.4)	11.585 (46.8)
≥50	3673 (54.2)	7824 (53.7)	1696 (49.6)	13 193 (53.2)
Gender^a^				
Female	2911 (43.0)	7886 (54.1)	1874 (54.8)	12 671 (51.1)
Male	3864 (57.0)	6694 (45.9)	1549 (45.2)	12 107 (48.9)
Self-reported race and ethnicity^a^				
Black	1502 (22.2)	3080 (21.1)	958 (28.0)	5540 (22.4)
Hispanic	688 (10.1)	985 (6.8)	193 (5.6)	1866 (7.5)
White	3962 (58.5)	9632 (66.1)	2045 (59.8)	15 639 (63.1)
Other races and ethnicities^c^	576 (8.5)	806 (5.5)	203 (5.9)	1585 (6.4)
Missing	47 (0.7)	77 (0.5)	24 (0.7)	148 (0.6)
Marital status^a^				
Married	2760 (40.7)	6683 (45.8)	1477 (43.1)	10 920 (44.1)
Not married	3973 (58.7)	7770 (53.3)	1913 (55.9)	13 656 (55.1)
Missing	42 (0.6)	127 (0.9)	33 (1.0)	202 (0.8)
Service-connected disability^a^				
Yes	4607 (68.0)	9914 (68.0)	2470 (72.2)	16 991 (68.6)
No	2168 (32.0)	4666 (32.0)	953 (27.8)	7787 (31.4)
Health insurance^a^				
Yes	1934 (28.5)	4770 (32.7)	1062 (31.0)	7766 (31.3)
No	4841 (71.5)	9810 (67.3)	2361 (69.0)	17 012 (68.7)
Cancer procedures in 2 y, No.^b^				
0	3568 (52.7)	7841 (53.8)	1887 (55.1)	13 296 (53.7)
1	1972 (29.1)	4389 (30.1)	990 (28.9)	7351 (29.7)
≥2	1235 (18.2)	2350 (16.1)	546 (16.0)	4131 (16.1)

^a^ All differences in category were statistically significant at *P* < .001.

^b^ All differences in category were statistically significant at *P* = .001.

^c^ American Indian or Alaskan Native, Asian, Native Hawaiian or other Pacific Islander, and unknown.

### Consultation Characteristics

Overall, 17 597 of 24 778 patients (71.0%) completed their genetic consultation (Table 2). However, only 1961 of 3423 patients (57.3%) completed their consultation if referred to non-VA care compared with 5073 of 6775 patients (74.9%) with the VA-traditional model and 10 563 of 14 580 patients (72.5%) with the VA-telehealth model, (*P* < .001). The median time for completion of a consultation referred to non-VA care was almost 3 times longer than observed in either VA model (median days for non-VA, 140 [IQR, 81-230] vs 55 days [IQR, 27-94] for the VA-traditional model and 45 days [IQR, 18-73] for the VA-telehealth model) (Table 2). The volume of referrals to all 3 models grew substantially with larger proportions received in later years (ie, 2016-2017) for VA-telehealth and non-VA care compared with the VA-traditional model. Most patients (13 951 of 14 580 [95.7%]) referred to the VA-telehealth model were from a different VA facility, whereas the VA-traditional model had only 2585 of 6775 (38.2%) interfacility referrals. Cancer was the most common referral reason (15 438 of 24 778 [62.3%]). However, the proportion of cancer referrals to the VA-traditional model was significantly smaller than the VA-telehealth model and non-VA care.

**Table 2.** Characteristics of Genetic Consultation Referrals by Type of Genetic Health Care Model

Consultation characteristics	Patient, No. (%)			
	VA-traditional model (n = 6775)	VA-telehealth model (n = 14 580)	Non-VA care (n = 3423)	Total (n = 24 778)
Complete^a^				
Yes	5073 (74.9)	10 563 (72.5)	1961 (57.3)	17 597 (71.0)
No	1702 (25.1)	4017 (27.5)	1462 (42.7)	7181 (29.0)
Reason for referral^a^				
Cancer^b^	3758 (55.5)	9581 (65.7)	2099 (61.3)	15 438 (62.3)
Gastrointestinal or polyposis	708 (10.5)	947 (6.5)	239 (7.0)	1894 (7.6)
Neurological or psychiatric disorders	524 (7.7)	1008 (6.9)	234 (6.8)	1766 (7.1)
Cardiovascular or connective tissue	439 (6.5)	612 (4.2)	159 (4.7)	1210 (4.9)
Other reasons^c^	1346 (19.9)	2432 (16.7)	692 (20.2)	4470 (18.0)
Year of referral^a^				
2010-2011	899 (13.3)	66 (0.5)	184 (5.4)	1149 (4.7)
2012-2013	1350 (19.9)	2156 (14.8)	442 (12.9)	3948 (15.9)
2014-2015	2017 (29.8)	5724 (39.3)	785 (22.9)	8526 (34.4)
2016-2017	2509 (37.0)	6634 (45.5)	2012 (58.8)	11 155 (45.0)
Setting^a^				
On-site	4190 (61.9)	629 (4.3)	0	4819 (19.5)
Interfacility	2585 (38.2)	13 951 (95.7)	0	16 536 (66.7)
Community	0	0	3423 (100.0)	3423 (13.8)
Days to complete, median (IQR)	55 (27-94)	45 (18,73)	140 (81 230)	53 (24-96)

Abbreviations: VA, Department of Veterans Affairs.

^a^ All differences in category were statistically significant at *P* < .001.

^b^ Cancer, patients affected with cancer and unaffected with family history of cancer, or unknown family history.

^c^ Allergy-immunology, congenital disorder (including chromosomal disorders), endocrine or metabolic, hematology, nephrology or urology, personal use (eg, ancestry, paternity), pharmacogenetics or exposures, pulmonary, reproductive concerns, rheumatology or autoimmune disorders, and not specified (eg, genetic counseling, genetic screening, positive genetic finding).

### Factors Associated With Type of Genetic Consultation Referral 

Compared with the VA-traditional model, women were 50% more likely referred to the VA-telehealth model (odds ratio [OR], 1.52; 95% CI, 1.21-1.92; *P* < .001), especially Black women, and patients of other races and ethnicities (American Indian/Alaskan Native, Asian, Native Hawaiian or other Pacific Islander, and individuals with unknown race or ethnicity) were about 50% less likely referred to the VA-telehealth model (OR, 0.54; 95% CI, 0.35-0.84; *P* = .006) (Table 3). Compared with the VA-traditional model, women were also 50% more likely to be referred to non-VA care (OR, 1.51; 95% CI, 1.09-2.09; *P* = .01), and Hispanic patients were about 50% less likely to be referred to non-VA care (OR, 0.52; 95% CI, 0.31-0.87; *P* = .01). Age, having a service-connected disability, having health insurance, and reason for referral were not significantly associated with referral to the VA-telehealth model or non-VA care.

**Table 3.** Patient and Consultation Characteristics Associated With Type of Genetic Consultation Referral

Variables	VA-telehealth model (ref: VA-traditional model)				Non-VA care (ref: VA-traditional model)			
	Model 1^a^		Model 2^b^		Model 1^a^		Model 2^b^	
	OR (95% CI)	*P* value	OR (95% CI)	*P* value	OR (95% CI)	*P* value	OR (95% CI)	*P* value
Age ≥50 y (REF: <50)	1.06 (0.90-1.24)	.50	1.08 (0.85-1.39)	.52	0.94 (0.76-1.17)	.58	0.86 (0.61-1.21)	.38
Female (REF: male)	1.52 (1.21-1.92)	<.001	1.41 (1.08-1.84)	.01	1.51 (1.09-2.09)	.01	1.29 (0.86-1.91)	.22
Age ≥50 × female (REF: age <50 × female or male)	NA	NA	0.96 (0.74-1.25)	.78	NA	NA	1.18 (0.84-1.66)	.34
Black race (REF: White race)	0.80 (0.46-1.39)	.43	0.63 (0.36-1.06)	.08	1.16 (0.66-2.04)	.61	1.05 (0.55-2.02)	.87
Black race × female (REF: White female or male)	NA	NA	1.56 (1.24-1.98)	<.001	NA	NA	1.25 (0.78-1.98)	.35
Hispanic ethnicity (REF: White race)	0.57 (0.31-1.08)	.08	0.60 (0.23-1.56)	.29	0.52 (0.31-0.87)	.01	0.47 (0.26-0.88)	.02
Hispanic ethnicity × female (REF: White female or male)	NA	NA	0.94 (0.40-2.22)	.88	NA	NA	1.20 (0.81-1.77)	.38
Other races or ethnicities^c^ (REF: White race)	0.54 (0.35-0.84)	.006	0.50 (0.29-0.85)	.01	0.63 (0.38-1.04)	.07	0.55 (0.28-1.07)	.08
Other races × female (REF: White female or male)	NA	NA	1.18 (0.84-1.66)	.33	NA	NA	1.30 (0.79-2.14)	.31
Married (REF: not married)	1.28 (1.01-1.61)	.04	1.28 (1.01-1.62)	.04	1.17 (0.99-1.49)	.19	1.18 (0.92-1.51)	.19
Service-connected disability (REF: not svc-connected)	0.96 (0.80-1.14)	.64	0.96 (0.80-1.14)	.62	1.11 (0.91-1.36)	.30	1.11 (0.91-1.35)	.31
Health insurance (REF: no health insurance)	1.20 (0.99-1.47)	.06	1.20 (0.98-1.46)	.07	1.15 (0.90-1.46)	.26	1.15 (0.90-1.46)	.27
Cancer referral^d^ (REF: other reasons^e^)	1.28 (0.97-1.69)	.08	1.27 (0.96-1.68)	.10	0.99 (0.64-1.53)	.97	0.99 (0.64-1.54)	.97
Gastrointestinal or polyposis referral (REF: other^e^)	0.77 (0.48-1.24)	.28	0.76 (0.47-1.23)	.26	0.75 (0.42-1.34)	.33	0.76 (0.42-1.36)	.36
Neurological/psychiatric referral (REF: other^e^)	1.10 (0.84-1.44)	.48	1.09 (0.83-1.42)	.53	0.94 (0.59-1.50)	.80	0.94 (0.59-1.50)	.80
Cardiovascular or CTD referral (REF: other^e^)	0.79 (0.58-1.10)	.17	0.81 (0.58-1.12)	.20	0.72 (0.43-1.19)	.20	0.72 (0.44-1.20)	.21

Abbreviations: CTD, connective tissue disorder; NA, not applicable; VA, Department of Veterans Affairs.

^a^ Model 1, main effects only.

^b^ Model 2, main effects with age and race or ethnicity × gender interactions.

^c^ American Indian or Alaskan Native, Asian, Native Hawaiian or other Pacific Islander, and unknown.

^d^ Patients affected with cancer and unaffected with family history of cancer.

^e^ Allergy-immunology, congenital disorder (including chromosomal disorders), endocrine/metabolic, hematology, nephrology/urology, personal utility (eg, ancestry, paternity), pharmacogenetics/exposures, pulmonary, reproductive concerns, rheumatology/autoimmune disorders, and not specified (eg, genetic counseling, genetic screening, positive genetic finding).

### Completed Genetic Consultations

There were 5073 of 6775 consultations (74.9%) completed with the VA-traditional model that were conducted in person (2638 [52%]), by telephone (1218 [24%]), by clinician-to-clinician e-consultations (710 [14%]), and video-telehealth, (507 [10%]). Video-telehealth was the predominant delivery mode (7606 [72%]) for the 10 563 consultations completed with the VA-telehealth model, followed by e-consultations (2535 [24%]), in-person (211 [2%]), and telephone (211 [2%]). We assumed all 1961 consultations completed through non-VA care were in person since other delivery modes were not possible or not reimbursed for non-VA care during the study period. We determined clinician types completing the consultation for 4487 of 5073 (88%) of the VA-traditional consultations and 9807 of 10 563 (93%) of the VA-telehealth consultations. For the VA-traditional model, 2423 (54%) were completed by geneticists, 538 (12%) by genetic counselors, and 1526 (34%) by both. The VA-telehealth model consultations were completed mostly by genetic counselors (9709 of 9807 [99%]), with only (98 of 9807 [1%]) completed by a geneticist, and 0 completed by both.

### Factors Associated With Completing Genetic Consultations

Patient and consultation characteristics associated with completing genetic consultations are included in Table 4. Patients referred to non-VA care compared with the VA-traditional model were 55% less likely to complete their genetic consultations (OR, 0.45; 95% CI, 0.35-0.57; *P* < .001). Older patients were more likely to complete their genetic consultations (OR, 1.12; 95% CI, 1.03-1.21; *P* = .01), but this association became insignificant when moderating effects of age on gender or age on care model were included. Black patients (OR, 0.84, 95% CI, 0.76-0.95; *P* = .001) and patients of other races and ethnicities (OR, 0.85; 95% CI, 0.76-0.96; *P* = .01) were less likely to complete their consultations compared with White patients. However, when moderating effects of care model type on race and ethnicity were included, the negative association for Black patients was only observed for those referred to the VA-telehealth model, and the negative association for patients of other races and ethnicities was only observed for those referred to non-VA care. Hispanic patients referred to non-VA care were also less likely to complete their genetic consultation.

**Table 4.** Patient and Consultation Characteristics Associated With Completing a Genetic Consultation

Variables	Model 1^a^		Model 2^b^		Model 3^c^	
	OR (95% CI)	*P* value	OR (95% CI)	*P* value	OR (95% CI)	*P* value
Age ≥50 (REF: age <50)	1.12 (1.03-1.21)	.01	1.04 (0.92-1.17)	.53	1.13 (0.99-1.29)	.07
Female (REF: male)	1.01 (0.90-1.31)	.91	0.94 (0.83-1.07)	.37	1.03 (0.84-1.26)	.78
Age ≥50 × female (REF: age <50 × female or male)	NA	NA	1.13 (0.98-1.31)	.09	NA	NA
Black race (REF: White race)	0.84 (0.76-0.93)	.001	0.78 (0.67-0.90)	.001	0.99 (0.86-1.14)	.88
Black race × female (REF: White race × female or male)	NA	NA	1.12 (0.92-1.37)	.25	NA	NA
Hispanic ethnicity (ref: White race)	1.08 (.80-1.46)	.62	1.33 (0.77-2.31)	.30	1.11 (0.94-1.30)	.21
Hispanic ethnicity × female (REF: White race × female or male)	NA	NA	0.68 (0.39-1.17)	.16	NA	NA
Other races or ethnicities^d^ (REF: White race)	0.85 (0.76-0.96)	.01	0.83 (0.72-0.95)	.007	0.93 (0.76-1.13)	.47
Other races × female (REF: White race × female or male)	NA	NA	1.05 (0.81-1.37	.70	NA	NA
Married (REF: not married)	1.03 (0.96-1.11)	.42	1.04 (0.96-1.12)	.37	1.03 (0.96-1.11)	.44
Service-connected disability (REF: none)	1.28 (1.16-1.41)	<.001	1.28 (1.16-1.41)	<.001	1.28 (1.16-1.41)	<.001
Health insurance (REF: no health insurance)	1.41 (1.30-1.52)	<.001	1.41 (1.30-1.52	<.001	1.41 (1.30-1.52	<.001
Cancer referral^e^ (REF: other referral reasons^f^)	0.95 (0.85-1.06)	.38	0.95 (0.86-1.06)	.38	0.95 (0.85-1.05)	.33
Gastrointestinal or polyposis referral (REF: other referral reasons^f^)	0.93 (0.79-1.10)	.40	0.95 (0.80-1.12)	.55	0.93 (0.79-1.10)	.39
Cardiovascular or CTD referral (REF: other referral reasons^f^)	1.02 (0.80-1.31)	.87	1.03 (0.80-1.31)	.83	1.01 (0.79-1.30)	.94
Neurological or psychiatric referral (REF: other referral reasons^f^)	1.01 (0.89-1.15)	.85	1.02 (0.89-1.16)	.82	1.01 (0.88-1.15)	.94
VA-Telehealth model (REF: VA-traditional model)	0.87 (0.75-1.01)	.06	0.87 (0.75-1.00)	.06	0.98 (0.81-1.19)	.87
VA-TH ×						
Age ≥50 (REF: VA-TH × age <50 or VA-traditional)	NA	NA	NA	NA	1.00 (0.85-1.19)	.96
Female (REF: VA-TH × male or VA-traditional)	NA	NA	NA	NA	0.91 (0.73-1.13)	.40
Black (REF: VA-TH × White race or VA-traditional)	NA	NA	NA	NA	0.74 (0.62-0.89)	.001
Hispanic (REF: VA-TH × White race or VA-traditional)	NA	NA	NA	NA	1.04 (0.59-1.83)	.89
Other races^d^ (REF: VA-TH × White race or VA-traditional)	NA	NA	NA	NA	0.96 (0.76-1.21)	.71
Non-VA care (REF: VA-traditional model)	0.45 (0.35-0.57)	<.001	0.45 (0.34-0.57)	<.001	0.43 (0.28-0.65)	<.001
Non-VA ×						
Age ≥50 (REF: Non-VA × age <50 or VA-traditional)	NA	NA	NA	NA	0.89 (0.68-1.17)	.41
Female (REF: Non-VA × male or VA-traditional)	NA	NA	NA	NA	1.25 (0.89-1.74)	.20
Black race (REF: Non-VA × White race or VA-traditional)	NA	NA	NA	NA	1.04 (0.80-1.34)	.80
Hispanic ethnicity (REF: Non-VA × White race or VA-traditional)	NA	NA	NA	NA	0.72 (0.57-0.91)	.005
Other races^d^ (REF: Non-VA × White race or VA-traditional)	NA	NA	NA	NA	0.68 (0.49-0.94)	.02

Abbreviations: CTD, connective tissue disorder; NA, not applicable; OR, odds ratio; REF, referent group; TH, telehealth; VA, Department of Veterans Affairs.

^a^ Model 1, main effects only.

^b^ Model 2, main effects with age and race or ethnicity × gender interactions.

^c^ Model 3, main effects with care model × age, race or ethnicity, and gender interactions.

^d^ American Indian or Alaskan Native, Asian, Native Hawaiian or other Pacific Islander, and unknown.

^e^ Patients affected with cancer and unaffected with family history of cancer.

^f^ Allergy-immunology, congenital disorder (including chromosomal disorders), endocrine or metabolic, hematology, nephrology or urology, personal utility (eg, ancestry, paternity), pharmacogenetics or exposures, pulmonary, reproductive concerns, rheumatology or autoimmune disorders, and not specified (eg, genetic counseling, genetic screening, positive genetic finding).

### Factors Associated With Multiple Cancer Surveillance or Risk-Reducing Procedures

Patient and consultation characteristics associated with cancer surveillance and risk-reducig procedures within 2 years of genetics referral are included in Table 5. Older patients and women had twice the odds of multiple surveillance or risk-reducing procedures in the VA 2 years following their referral (older patients: OR, 2.00; 95% CI, 1.85-2.15; *P* < .001; women: OR, 2.00; 95% CI, 1.72-2.32; *P* < .001). Although with moderating effects of age on gender, older women were less likely to have multiple procedures. Black patients and Hispanic patients had greater odds of multiple procedures than White patients (Black patients: OR, 1.26; 95% CI, 1.11-1.44; *P* = .001; Hispanic patients: OR, 1.24; 95% CI, 1.06-1.45; *P* = .008). Although with moderating effects of race and ethnicity on gender, the association reversed direction for Hispanic females. Patients referred for cancer or GI conditions and polyposis had greater odds of multiple procedures than patients referred for other reasons (cancer: OR, 1.70; 95% CI, 1.54-1.89; *P* < .001; GI conditions and polyposis: OR, 2.89; 95% CI, 2.29-3.65; *P* < .001); whereas patients referred for neurological or psychiatric disorders had lesser odds (OR, 0.76; 95% CI, 0.66-0.88; *P* < .001). Patients completing consultations were 55% more likely to have multiple cancer surveillance and risk-reducing procedures (OR, 1.55; 95% CI, 1.40-1.72; *P* < .001). However, with moderating effects of care model by consultation completion status, the direction of the association reversed for the VA-telehealth model and non-VA care, with both becoming negative.

**Table 5.** Patient and Consultation Characteristics Associated With Cancer Surveillance and Risk-Reducing Procedures Within 2 Years of Genetics Referral

Variables	Model 1^a^		Model 2^b^		Model 3^c^		Model 4^d^	
	OR (95% CI)	*P* value	OR (95% CI)	*P* value	OR (95% CI)	*P* value	OR (95% CI)	*P* value
Age >50 (REF: age <50)	2.00 (1.85-2.15)	<.001	2.28 (2.07-2.52)	<.001	2.18 (1.86-2.56)	<.001	2.00 (1.85-2.15)	<.001
Women (REF: men)	2.00 (1.72-2.32)	<.001	2.26 (1.94-2.65)	<.001	2.25 (1.75-2.88)	<.001	2.00 (1.72-2.32)	<.001
Age ≥50 × female (REF: age <50 × female or male)	NA	NA	0.81 (0.71-0.91)	.001	NA	NA	NA	NA
Black race (REF: White race)	1.26 (1.11-1.44)	.001	1.17 (1.04-1.32)	.010	1.13 (0.95-1.35)	.17	1.26 (1.10-1.43)	.001
Black race × women (REF: Black men or White race)	NA	NA	1.12 (0.92-1.36)	.27	NA	NA	NA	NA
Hispanic ethnicity (REF: White race)	1.24 (1.06-1.45)	.008	1.44 (1.22-1.70)	<.001	1.10 (0.96-1.27)	.16	1.24 (1.05-1.46)	.01
Hispanic ethnicity × female (REF: Hispanic male or White race)	NA	NA	0.75 (0.61-0.92)	.005	NA	NA	NA	NA
Other races or ethnicities^e^ (REF: White race)	0.86 (0.76-0.96)	.009	0.78 (0.66-0.92)	.004	0.80 (0.67-0.96)	.02	0.86 (0.76-0.96)	.008
Other races^e^ × female (REF: other races^e^ × male or White race)	NA	NA	1.18 (0.97-1.44)	.10	NA	NA	NA	NA
Married (REF: not married)	0.95 (0.90-1.00)	.06	0.94 (0.89-1.00)	.04	0.95 (0.90-1.00)	.06	0.95 (0.90-1.00)	.07
Service-connected disability (REF: none)	1.07 (1.01-1.14)	.03	1.07 (1.01-1.14)	.02	1.07 (1.01-1.14)	.02	1.07 (1.01-1.13)	.03
Health insurance (REF: no health insurance)	0.99 (0.94-1.05)	.82	0.99 (0.93-1.05)	.68	0.99 (0.94-1.05)	.79	1.00 (0.94-1.06)	.90
Cancer referral^f^ (REF: other referral reasons^g^)	1.70 (1.54-1.89)	<.001	1.69 (1.52-1.87)	<.001	1.69 (1.53-1.88)	<.001	1.71 (1.54-1.89)	<.001
Gastrointestinal or polyposis referral (REF: Other referral reasons^g^)	2.89 (2.29-3.65)	<.001	2.83 (2.24-3.57)	<.001	2.88 (2.28-3.64)	<.001	2.90 (2.30-3.66)	<.001
Neurological or psychiatric referral (REF: other referral reasons^g^)	0.76 (0.66-0.88)	<.001	0.75 (0.65-0.87)	<.001	0.76 (0.66-0.87)	<.001	0.76 (0.66-0.87)	<.001
Cardiovascular or CTD referral (REF: Other referral reasons^g^)	0.92 (0.77-1.10)	.38	0.93 (0.78-1.11)	.42	0.93 (0.78-1.11)	.41	0.92 (0.77-1.10)	.35
Consultation completed (REF: not completed)	1.55 (1.40-1.72)	<.001	1.55 (1.40-1.72)	<.001	1.55 (1.40-1.72)	<.001	2.14 (1.81-2.53)	<.001
VA-TH × completed (REF: completed × VA-traditional or not)	NA	NA	NA	NA	NA	NA	0.66 (0.56-0.78)	<.001
Non-VA × completed (REF: completed × VA-traditional or not)	NA	NA	NA	NA	NA	NA	0.64 (0.52-0.78)	<.001
VA-Telehealth model (REF: VA-traditional model)	0.86 (0.72-1.03)	.10	0.86 (0.72-1.02)	.09	0.95 (0.80-1.13)	.58	1.19 (0.99-1.42)	.06
VA-TH ×								
Age ≥50 (REF: VA-TH and <50 or VA-traditional)	NA	NA	NA	NA	0.86 (0.72-1.04)	.12	NA	NA
Women (REF: VA-TH and men or VA-traditional)	NA	NA	NA	NA	0.85 (0.64-1.14)	.64	NA	NA
Black race (REF: VA-TH and White race or VA-traditional)	NA	NA	NA	NA	1.18 (0.93-1.49)	.17	NA	NA
Hispanic (REF: VA-TH and White race or VA-traditional)	NA	NA	NA	NA	1.25 (0.96-1.61)	.10	NA	NA
Other races^e^ (REF: VA-TH and White race or VA-traditional)	NA	NA	NA	NA	1.10 (0.87-1.38)	.43	NA	NA
Non-VA care (REF: VA-traditional model)	0.89 (0.73-1.08)	.23	0.89 (0.73-1.08)	.23	0.95 (0.75-1.21)	.68	1.23 (1.02-1.49)	.03
Non-VA ×								
Age ≥50 (REF: non-VA × age <50 or VA-traditional)	NA	NA	NA	NA	0.97 (0.78-1.22)	.81	NA	NA
Female (REF: non-VA × male or VA-traditional)	NA	NA	NA	NA	0.85 (0.57-1.26)	.41	NA	NA
Black (REF: non-VA × White race or VA-traditional)	NA	NA	NA	NA	1.10 (0.85-1.42)	.48	NA	NA
Hispanic (REF: non-VA × White race or VA-traditional)	NA	NA	NA	NA	0.95 (0.70-1.29)	.74	NA	NA
Other races^e^ (REF: non-VA × White race or VA-traditional)	NA	NA	NA	NA	1.14 (0.83-1.58)	.42	NA	NA

Abbreviations: CTD, connective tissue disorder; NA, not applicable; OR, odds ratio; REF, referent group; TH, telehealth; VA, Department of Veterans Affairs.

^a^ Model 1, main effects only.

^b^ Model 2, main effects with age and race or ethnicity × gender interactions.

^c^ Model 3, main effects with genetic care model × age, race or ethnicity and gender interactions.

^d^ Model 4, main effects with genetic care model × consultation status interactions; CVD, cardiovascular disease. Each model is comparing 0 vs 2 or more cancer surveillance and risk-reducing procedures.

^e^ American Indian or Alaskan Native, Asian, Native Hawaiian or other Pacific Islander, and unknown.

^f^ Patients affected with cancer and unaffected with family history of cancer.

^g^ Allergy immunology, congenital disorder (including chromosomal disorders), endocrine or metabolic, hematology, nephrology or urology, personal use (eg, ancestry, paternity), pharmacogenetics or exposures, pulmonary, reproductive concerns, rheumatology or autoimmune disorders, and not specified (eg, genetic counseling, genetic screening, positive genetic finding).

## Discussion

Like other studies comparing VA services and non-VA care,^18^ in this cross-sectional study, we found that the VA genetic care models—both traditional and telehealth—had better care coordination than non-VA care. The effectiveness and timeliness to complete genetic consultations was better with the VA. The VA-telehealth model was associated with improved access to VA genetic services, especially for cancer referrals, with greater capacity for growth compared with the VA-traditional model. This is likely because of efficiencies of scale given the centralized nature of the VA-telehealth model. Cancer genetic consultations typically result in recommendations for more cancer surveillance and risk-reducing procedures with greater patient adherence.^19,20^ Having multiple cancer surveillance and risk-reducing procedures was more likely if completing the genetic consultation with the VA-traditional model than VA-telehealth, which performed similarly to non-VA care. This suggests better care coordination or a greater influence of the VA-traditional model on patient uptake of these procedures. With most VA-telehealth model encounters conducted solely by genetic counselors, this could have constrained the recommendations that could be made for these procedures and the ability to directly order them, given the genetic counselors’ scope of practice.^21,22^

The VA-telehealth model was associated with exacerbated health care disparities based on race or ethnicity and gender compared with the VA-traditional model. In the VA, telehealth use is known to be lower in Asian patients, Black patients, and Hispanic patients compared with White patients.^9^ We observed this happening at the consultation referral stage for patients of Asian, American Indian or Alaskan Native, and Native Hawaiian or Pacific Islander ancestry, and at the consultation completion stage for Black patients. Biases of referring physicians regarding perceived preferences for patients of Alaskan Native or American Indian, Asian, and Native Hawaiian or Pacific Islander ancestry for VA-traditional genetic services may explain disparities at the referral stage. Alternatively, these patients may have declined referral to VA-telehealth when given options. Once referred for genetic consultation, Black patients were less likely to complete their consultation if referred to the VA-telehealth model. Studies have characterized the communication between Black patients and their health care professionals as emotionally less open,^23^ and Black patients rate their visits with their health care professionals as less participatory than White patients.^24^ Additionally, Black patients have greater mistrust of the medical community and more concern of potential misuse of genetic information because of historical experiences like the Tuskegee experiment and the racially motivated eugenics movement.^25,26^ Thus, Black patients may have perceived the VA-telehealth model as a barrier that further complicates patient-clinician communication. Effective communication by the referring clinician explaining the relevance of the genetic consultation to patient care may help alleviate patients’ concerns and ensure follow-through on the referral.

Black patients and Hispanic patients were more likely to have multiple cancer surveillance and risk-reducing procedures in the VA within 2 years of their genetic consultation referral than White patients. This may be due to more options for these procedures outside of the VA for White patients or preference to obtain these procedures within VA among Black patients and Hispanic patients. This observation contradicts literature describing disparities in health care utilization among racial and ethnic minorities in the VA and in the community.^27^ VA research has shown these disparities are most prevalent for care processes likely affected by the quantity and quality of patient-clinician communication, shared decision-making, and patient participation.^27^ Our results suggest VA genetics clinicians may be more adept at preparing patients for uptake of cancer preventive procedures, especially under the VA-traditional model.

Genetic services are highly relevant to women, with a substantial over-representation of women referred compared with men. Women were referred to the VA-telehealth model and non-VA care more than the VA-traditional model programs based at large VA medical centers. This is consistent with research from Northern California Kaiser-Permanente that has found women are more likely than men to choose a telemedicine visit.^28^ The greater likelihood of genetics referral to the VA-telehealth model or non-VA care may be partly because of a preference to avoid VA medical centers where the traditional models are based because of harassment experienced at certain VA facilities.^29^

Women were twice as likely to have multiple cancer surveillance and risk-reducing procedures in the VA 2 years following the genetics referral compared to men. This may be due in part to including procedures that are more relevant for women in our analysis (eg, mammogram, bilateral mastectomy). Additionally, men consistently underuse preventive health care services compared with women.^30,31^ Notably, while the VA-telehealth model appears to be preferred by women for genetic consultation, patients completing consultations with the VA-telehealth model were less likely to have multiple cancer surveillance and risk-reducing procedures in the VA. This may be due to geographic barriers that promote use of telehealth yet present barriers to obtaining these procedures in person at the VA, certain cancer-preventive procedures (eg, mammography) may not be available in the VA, or women may prefer obtaining these procedures in the community,^32^ which may be due in part to fear of harassment while seeking care at VA.^29^

Age was not associated with referral to the genetic care models or with completing consultations when the moderating effects of gender or care model were considered. Older patients were more likely to have multiple cancer-preventive procedures following a genetics referral, except older women were less likely. Older patients generally use more health care services than younger patients.^33^ Older patients are expected to have more cancer-preventive procedures because age 50 is a typical age for beginning cancer screening for average-risk patients in the VA.^34,35,36^ However, for high-risk patients, cancer surveillance and risk-reducing surgeries are typically recommended before age 50, and this may explain our findings of younger women having greater odds of multiple procedures.^18,37^

The disparities observed in completing genetic consultations and uptake of cancer surveillance and risk-reducing procedures based on the type of genetic care model are likely multidimensional and may be best explained by the centralized structure and uniform approach of the VA-telehealth model that limits patient-clinician encounters to the telehealth delivery mode performed almost exclusively by genetic counselors. Centralized services may improve efficiencies of operational and administrative processes but also can challenge care coordination by constraining the ability to tailor services to local needs, stifling initiative and innovation, and complicating communication processes between the staff, patients, and referring clinicians.^38,39^ Further, vulnerable subpopulations are less able to benefit from a centralized approach because of inconsistencies between the social and cultural assumptions of those implementing the approach and the targeted groups.^40^

Health care disparities associated with telehealth technology are attributed to the digital divide or lack of access to telehealth devices, software, and broadband internet.^41^ However, the digital divide cannot explain the disparities we observed in completing genetic consultations, since video-to-clinic encounters were used rather than video-to-home during the study period. Our findings suggest that shrinkage of the digital divide by improving access to telehealth equipment and the internet will not suffice to ensure health care equity. Health care organizations seeking to improve access to specialty care using a centralized telehealth service should notice the VA’s experience and develop implementation plans that include a health equity framework to assess disparities and identify mitigating strategies.^14,42^

### Limitations

The large data set permitted the assessment of multiple variables to identify significant associations and moderating effects on the outcomes of interest. However, the observational study design limits the findings to associations, and there may be confounding from unmeasured variables. To address this in part, we have adjusted for clustering of patients by referring facility. An important outcome was the use of cancer surveillance and risk-reducing procedures. However, we only assessed these procedures performed in the VA and not in the community. Thus, we may have underestimated use across the population studied and possibly within the different genetic care models. Another limitation is that the findings may not be generalizable. However, the VA is not unique in establishing a centralized telehealth model for delivering genetic services nationwide; multiple commercial entities and academic institutions offer similar services.

## Conclusions

In this cross-sectional study, we found that a centralized telehealth model was associated with access to genetic services but also with hindered care coordination for follow-up services and exacerbation of health care disparities. While there may be efficiencies of scale related to the centralized telehealth model, this must be balanced against other quality outcomes, such as effectiveness, patient-centeredness, and equity. As the need for genetic services continues to grow, the VA and other stakeholders relying on centralized telehealth services for specialty care must assess structural barriers and the needs and preferences of vulnerable subpopulations. The needs and preferences may complement the centralized approach, improve care coordination, and help mitigate health care disparities if these barriers are addressed.

## Notes

Supplement. **eAppendix.** Consult Reason Classification **eTable 1.** Confusion Matrix Comparing Predicted Class From NLP System to Annotator Opinion **eTable 2.** Scores
eReferences
