# Supplementary material for: Demographic Differences Among US Department of Veterans Affairs Patients Referred for Genetic Consultation to a Centralized VA Telehealth Program, VA Medical Centers, or the Community
Source: JAMA Netw Open. 2022 Apr 11;5(4):e226687. doi: 10.1001/jamanetworkopen.2022.6687 (PMC9002339; doi:10.1001/jamanetworkopen.2022.6687)
Supplement: Supplement. — eAppendix. Consult Reason Classification eTable 1. Confusion Matrix Comparing Predicted Class From NLP System to Annotator Opinion eTable 2. Scores eReferences [file jamanetwopen-e226687-s001.pdf]

Supplemental Online Content

Scheuner MT, Huynh AK, Chanfreau-Coffnier C, et al. Demographic differences among US Department of Veterans Affairs patients referred for genetic consultation to a centralized VA telehealth program, VA medical centers, or the community. *JAMA Netw Open*. 2022;5(4):e226687. doi:10.1001/jamanetworkopen.2022.6687

eAppendix. Consult Reason Classification

eTable 1. Confusion Matrix Comparing Predicted Class From NLP System to Annotator Opinion

eTable 2. Scores

eReferences

This supplemental material has been provided by the authors to give readers additional information about their work.

© 2022 Scheuner MT et al. *JAMA Network Open.*

eAppendix. Consult Reason Classification

1 Problem

The Natural Language Processing (NLP) system intends to classify a reason for referral for a genetic consult within the VA healthcare system.

1.1 Class Definitions

There are 14 classes defined in the problem. However, due to difficulty classifying imbalanced datasets where certain classes have far more examples than others, the scope of the problem was limited to final validation of 4 of the largest classes. Additionally, over the course of development, several classes have been merged to represent larger groups of related consult reasons.
An abbreviated definition of the consult reason classes follows. Each item on the list is the associated class name followed by component diseases and disorder groups.

Primary classes:
_ Cancer:
  Cancer: An abnormal mass of tissue that results when cells divide more than they should or do not die when they should.
_ Gastroenterology:
  Gastrointestinal: Digestive diseases are disorders of the digestive tract, which is sometimes called the gastrointestinal tract.
  Polyps: A colon polyp is a small clump of cells that forms on the lining of the colon.
_ Cardiovascular disease
  Cardiovascular: Relating to the circulatory system, which comprises the heart and blood vessels and carries nutrients and oxygen to the tissues of the body and removes carbon dioxide and other wastes from them.
  Connective Tissue Disease: Connective tissue disease refers to a group of disorders involving the protein-rich tissue that supports organs and other parts of the body.
  Orthopedic: Anything that is concerned with muscles, ligaments and joints is considered orthopedic.
  Musculoskeletal: Musculoskeletal disorders are soft-tissue injuries caused by sudden or sustained exposure to repetitive motion, force, vibration and awkward positions.
  Dermatological: Skin disorders that vary in symptoms and severity.
_ Neurology:
  Neurological: Neurological disorders are diseases of the brain, spine and the nerves that connect them.
  Psychological: Psychologists define a psychological disorder broadly as psychological dysfunction in an individual that is associated with distress or impairment and a reaction that is not culturally expected.
  Eye: Neurological vision disorders are caused by conditions affecting the nervous system.

Other classes:
_ Allergy/Immunology:
  Allergy: An allergy is a damaging immune response by the body to a substance, especially pollen, fur, a particular food or dust, to which it has become hypersensitive.
  Immunology: Includes the structure, function, and disorders of the immune system.
_ Birth defects:
  Birth Defect: A physical or biochemical abnormality that is present at birth and may be inherited or the result of environmental influence
  Chromosomal Disorder: An abnormal condition due to something unusual in an individual's chromosomes.
  Hereditary Disorder: A genetic disease is any disease that is caused by an abnormality in an individual's genome, the person's entire genetic makeup.
_ Endocrinology:
  Endocrine: Endocrine disorders involve an abnormality of one of the body's endocrine glands.
  Metabolic: Disorders involving abnormal metabolism.
_ Hematology:
  Hematology: Hematology is the study of blood in health and disease. It includes problems with the red blood cells, white blood cells, platelets, blood vessels, bone marrow, lymph nodes, spleen, and the proteins involved in bleeding and clotting (hemostasis and thrombosis).
_ Nephrology:

© 2022 Scheuner MT et al. *JAMA Network Open.*

Genitourinary: Genitourinary refers to the urinary and genital organs.
Nephrology: Nephrology is the branch of medicine concerned with the kidney.

_ Personal utility:

Ancestry: Ancestry is comprising of a line of descent or lineage; ancestors. Genealogy is commonly used to identify one's ancestry.
Paternity: Paternity is the state of being someone's father. Paternal origin is relating to the origin or descent from a father/male.
Transgender: Transgender is relating to, or being a person, whose gender identity differs from the sex the person had or was identified as having at birth.

_ Pharmacogenetics:

Pharmacogenetics: Clinical pharmacogenetics is the use of genetic data to guide drug therapy decisions. It involves variations in drug response due to genetic makeup and studies how a person's genetic makeup can affect their metabolism of a drug.
Exposure: Exposures that might affect health, e.g., toxin, radiation, herbicide, pesticide, insecticide

_ Pulmonary:

Pulmonary: Any problem in the lungs that prevents the lungs from working properly. Affect the airways by causing a narrowing or blockage. Affect the structure of the lung tissue due to scarring or inflammation resulting in the lungs being unable to expand fully. Affect the pulmonary blood vessels.

_ Reproductive:

Reproductive: Reproductive health refers to the diseases, disorders and conditions that affect the functioning of the male and female reproductive systems during all stages of life.

_Rheumatology

Rheumatology: Immune-mediated disorders of the musculoskeletal system, soft tissues, blood vessels and includes autoimmune disorders

_ Not Specified:

A consult that does not fit any of the reasons described above, or no reason is specified in the note.

2 Method

To classify documents, we developed an iterative approach of classification, validation, and data augmentation to produce accurate results for Cancer, Gastroenterology, Cardiovascular disease, and Neurology classes.

2.1 Text Filtering

A critical component of document classification was eliminating irrelevant text in the document before any other processing is performed. Many documents contained standardized text that could be filtered out through manual creation of Regular Expressions and Filtering on a match. Filtering out information such as sending/receiving facilities, dates, form text (e.g., “Please enter the information below"), and other elements helped reduce the amount of text processed by other components of the algorithm.

Most importantly, this component of the software filters out lists of topic-relevant terms that are irrelevant for a particular patient or document. Text like “Enter patient family history of cancer:" is irrelevant to a particular consult reason but is common in documents and are removed during preprocessing. Additionally, terms with ambiguous meaning such as "CA" as an abbreviation for both "California" and "cancer" could be removed when an address was seen in text.

2.2 Pointwise Mutual Information

Pointwise Mutual Information (pmi) is a metric for measuring co-occurrence of two discrete random variables. The value of $\mathrm{pmi}(x; y)$ changes based on how much the observed probability of co-occurrence, $p(x; y)$, differs from the expected probability of co-occurrence assuming x and y are statistically independent, where $p(x; y) = p(x)p(y)$.

$$\mathrm{pmi}(x; y) = \ln p(x; y) / p(x)p(y)$$

When x and y are independent, then $\mathrm{pmi}(x; y) = 0$. If there is a negative association between x and y then $\mathrm{pmi}(x; y) < 0$. If there is a positive association between x and y then $\mathrm{pmi}(x; y) > 0$. The larger these associations, the more the values deviate from 0. This can be applied to a classification task by calculating pmi values between words in a document and a classification for that document as a whole. For every combination of word w and class c, we

© 2022 Scheuner MT et al. *JAMA Network Open.*

calculate the pmi(*w; c)*. For example, this creates a list of pmi values for the word “BRCA" and every potential classification (“CA", “GI", etc.) as well as the pmi value for every unique word and the “CA" class.

2.3 Stopword Curation

Stopwords in natural language processing are words that are filtered out before processing data. These are generally the most common words in the text or are words that do not contain meaning for the particular task. As part of development, a list of stopwords was manually curated as exclusions to the pmi calculations.

Due to the varied text in documents, there was a large amount of noise in the pmi results. Words that should not contain information relevant to the genetic consult reason often had pmi values that deviated far from 0. A few examples include “consult," “expected," “history," and “result." These words do not indicate a particular consult reason but may have surfaced disproportionately in one or more classifications by chance.

2.4 Document Processing and Classification

To produce output, each document was first filtered to remove any form data and irrelevant content. After filtering, the remaining text was broken down into a sequence of words. For each word in the sequence, the pmi scores were looked up and summed for each class with out-of-vocabulary words given a score of 0. The two classes with the highest scores were output with the associated document into the database tables. If all classes had a score of 0, then the “not specified" class is output.

2.5 Validation

To validate the system output, documents were selected at random from each predicted class and sent to annotators for expert classification.

2.6 Data Augmentation

After each round of validation, the annotated documents were added to the pool of training data and pmi scores were recalculated. The first round began using a list of the provisional diagnosis text manually classified into the correct class as initial training data. The primary goal of this was to improve the size of the vocabulary of the pmi values.

Because each class is so broad and contains many different mentions of diseases and genes (many of which would not have been seen in previous iterations), adding more training data decreased the total number of words in each document that must be given a score of 0.

3 Results

3.1 Class Distribution

The following is the distribution of predicted labels by the NLP system. Primary classes are bolded.

_ **Cancer**: 18,628
_ **Neurology**: 2,243
_ **Gastroenterology**: 2,213
_ Reproductive: 1,829
_ **Cardiovascular disease**: 1,569
_ Endocrinology: 737
_ Not specified: 592
_ Hematology: 587
_ Pharmacogenetics: 393
_ Nephrology: 339
_ Birth defect: 269
_ Pulmonary: 211
_ Allergy/Immunology: 115
_ Personal utility: 102

There are an additional 352 documents with the special category “NONE" that represent documents that contain no text after filtering out form text. These documents may be considered “Not Specified" but are not output by the system as such.

© 2022 Scheuner MT et al. *JAMA Network Open.*

3.2 Final Validation

30 documents were sampled from each of the primary classes (Cancer, Cardiovascular, Gastroenterology, Neurology) for the final validation below. The chart below shows a confusion matrix that compares predicted class from the NLP system to the annotator opinion. Confusion between a predicted “Cancer" class and a validated “Gastroenterology” class is the largest single point of error in the system. Most of these errors are caused by ambiguity in a diagnosis or family history of colon cancers.

© 2022 Scheuner MT et al. *JAMA Network Open.*

eTable 1. Confusion Matrix Comparing Predicted Class From NLP System to Annotator Opinion

		Prediction/NLP						
**Validation/Annotation**		**Allergy**	**CA**	**Cardio**	**Endo**	**GI**	**Heme**	**Neuro**
	**Allergy**	0	0	1	0	0	0	1
	**CA**	0	30	1	0	5	0	2
	**Cardio**	0	0	26	0	0	0	1
	**Endo**	0	0	1	0	0	0	0
	**GI**	0	0	0	0	25	0	1
	**Heme**	0	0	1	0	0	0	0
	**Neuro**	0	0	0	0	0	0	25

Three performance metrics are typically used when evaluating an NLP tool's performance: precision, recall, and F1. All scores have a range of [0,1] and are calculated using the True Positive (TP), False Positive (FP), and False Negative (FN) counts for each class. These counts are derived from the confusion matrix above.

3.2.1 Precision

Precision, also known as Positive Predictive Value (PPV), represents the fraction of documents in a predicted class that are correctly classified. It is the ratio of true positive classifications to all predicted instances of a class. Precision for the “CA" class will be lower when documents are incorrectly labeled as “CA".

$$Precision = TP / TP + FP$$

3.2.2 Recall

Recall, also known as Sensitivity, represents the fraction of documents correctly classified to all documents of a particular class. It is the ratio of true positive classifications to all instances of the class (which includes false negative classifications). Recall will be lower when the number of documents correctly classified “CA" is lower than the number of all “CA" documents.

$$Recall = TP / TP + FN$$

3.2.3 F1 Score

Precision and recall alone have several drawbacks. Recall for “CA" is 1, a perfect score, when every document is classified as “CA" regardless of correctness, because all real \CA" documents are correct. Precision for “CA" is 1 when only a single document is classified as “CA" because all classifications are correct, even if most real “CA" documents are ignored.

To combat these drawbacks, a harmonic mean of precision and recall, called the F1 score, is also reported. F1 is a value that is close to the average between precision and recall when the precision and recall are similar but results in a low score when precision and recall are very different from one another. This encourages balancing over- and under-classification of documents for every class measured.

$$F1 = 2 \text{ x } Precision \text{ x } Recall / Precision + Recall$$

eTable 2. Scores

The table below shows the precision, recall and F1 for the primary classes. All primary classes maintain an F1 score of around 0.9.

Primary Classes	Precision	Recall	F1
**Cancer**	1	0.78	0.88
**Cardiovascular**	0.87	0.96	0.91
**Gastroenterology**	0.83	0.96	0.89

© 2022 Scheuner MT et al. *JAMA Network Open.*

**Neurology**	0.83	1	0.91

eReferences

Church KW, Hanks P. Word association norms, mutual information, and lexicography. Comput Linguist, 1990;16(1):22-29.

Turney PD. Thumbs up or thumbs down?: Semantic orientation applied to unsupervised classification of reviews. In Proceedings of the 40th Annual Meeting on Association for Computational Linguistics, ACL '02, pages 417{424, Stroudsburg, PA, USA, 2002. Association for Computational Linguistics.

eTable1

Observed characteristics between participants with complete and incomplete data

Patient characteristics	Complete data n=24,432	Incomplete data n=346	Total n=24,778
Mean age, years (SD)	50.7 (14.8)	44.0 (15.8)	50.6 (14.9)
Age groups, No. (%)			
<50	11,365 (46.5)	220 (63.6)	11.585 (46.8)
>/=50	13,067 (53.5)	126 (36.4)	13,193 (53.2)
Gender, No. (%)			
Female	12,464 (51.0)	207 (59.8)	12,671 (51.1)
Male	11,968 (49.0)	139 (40.2)	12,107 (48.9)
Self-reported race/ethnicity, No. (%)			
Black	5,499 (22.5)	41 (11.8)	5,540 (22.4)
Hispanic	1,849 (7.6)	17 (4.9)	1,866 (7.5)
White	15,536 (63.6)	103 (29.8)	15,639 (63.1)
Other races/ethnicities[a]	1,548 (6.3)	37 (10.7)	1,585 (6.4)
Missing	0 (0.0)	148 (42.8)	148 (0.6)
Marital status, No. (%)			
Married	10,848 (44.4)	72 (20.8)	10,920 (44.1)
Not married	13,584 (55.6)	72 (20.8)	13,656 (55.1)
Missing	0 (0.0)	202 (58.4)	202 (0.8)
Service-connected disability, No. (%)			
Yes	16,787 (68.7)	204 (59.0)	16,991 (68.6)
No	7,645 (31.3)	142 (41.0)	7,787 (31.4)
Health insurance, No. (%)			
Yes	7,677 (31.4)	89 (25.7)	7,766 (31.3)
No	16,755 (68.6)	257 (74.3)	17,012 (68.7)
# cancer procedures in 2 years, No. (%)			
0	13,093 (53.6)	203 (58.7)	13,296 (53.7)
1	7,252 (29.7)	99 (28.6)	7,351 (29.7)
2 or more	4,087 (16.7)	44 (12.7)	4,131 (16.6)

[a]American Indian or Alaskan Native, Asian, Native Hawaiian or Other Pacific Islander, and unknown

© 2022 Scheuner MT et al. *JAMA Network Open.*

eTable2

Differences in proportions of patient characteristics from the source populations for the VA-telehealth and VA-traditional models

Patient characteristics	Proportions (95% CI) in the VA-telehealth model source population (n=7,058,074)	Proportions (95% CI) in the VA-traditional model source population (n=1,050,855)	Difference in proportions (95% CI) between VA-telehealth source and VA-traditional source populations
Women	0.088 (0.0878, 0.0882)	0.089 (0.0885, 0.0895)	-0.001 (-0.0016, -0.0004)
Age groups, years			
<40	0.164 (0.1637, 0.1643)	0.204 (0.2032, 0.2048)	-0.040 (-0.0408, -0.0392)
40-64	0.436 (0.4356, 0.4364)	0.428 (0.4271, 0.4289)	0.008 (0.00070, 0.0090)
>/=65	0.400 (0.4000, 0.4004)	0.368 (0.3671, 0.3689)	0.032 (0.0310, 0.0330)
Race/ethnicity			
Black	0.146 (0.1457-0.1463)	0.1520 (0.1513, 0.1527)	-0.006 (-0.0067, -0.0053)
Hispanic	0.056 (0.0558-0.0562)	0.088 (0.0875, 0.0885)	-0.032 (-0.0326, -0.0314)
Other races [a]	0.131 (0.1308-0.1312)	0.146 (0.1453, 0.1467)	-0.015 (-0.0157, -0.0143)
White	0.667 (0.6667-0.6673)	0.615 (0.6141, 0.6159)	0.052 (0.0510, 0.0530)
Married	0.542 (0.5416-0.5424)	0.4850 (0.4840, 0.4860)	0.057 (0.0560, 0.0580)
Service-connected	0.539 (0.5386-0.5394)	0.578 (0.5771, 0.5789)	-0.039 (-0.0400, -0.0380)
Health insurance	0.501 (0.5006-0.5014)	0.435 (0.4341, 0.4359	0.066 (0.0650, 0.0670)

VA=Department of Veterans Affairs

[a] American Indian or Alaskan Native, Asian, Native Hawaiian or Other Pacific Islander

© 2022 Scheuner MT et al. *JAMA Network Open.*
